# Supplementary material for: Synthesis and Properties of NitroHPHAC: The First Example of Substitution Reaction on HPHAC
Source: Molecules. 2020 May 27;25(11):2486. doi: 10.3390/molecules25112486 (PMC7321187; doi:10.3390/molecules25112486)
Supplement: Supplementary file 1 [file molecules-25-02486-s001.pdf]

Supporting information

Synthesis and properties of nitroHPHAC: The first example of substitution reaction of HPHAC

Yoshiki Sasaki, Masayoshi Takase*, Shigeki Mori, and Hidemitsu Uno*

Graduate School of Science and Engineering, Ehime University, Matsuyama 790-8577, Japan

E-mail: takase.masayoshi.ry@ehime-u.ac.jp
E-mail: uno@ehime-u.ac.jp

Contents

1. NMR spectra p. S2
2. Mass spectra p. S4
3. IR spectra p. S6
4. Solvatochromism and dipole moments p. S8
5. X-ray crystal structures p. S9
6. Comparison of the ^{1}H-NMR spectra of **2a**, **2a**$^{2+}$ and **1a**$^{2+}$ p. S13
7. TD-DFT calculations p. S14
8. ACID plots and NICS calculations p. S19
9. Possible resonance effect of **2b** p. S20
10. Atomic coordinates of the optimized structures p. S21
11. References p. S25

1. NMR spectra

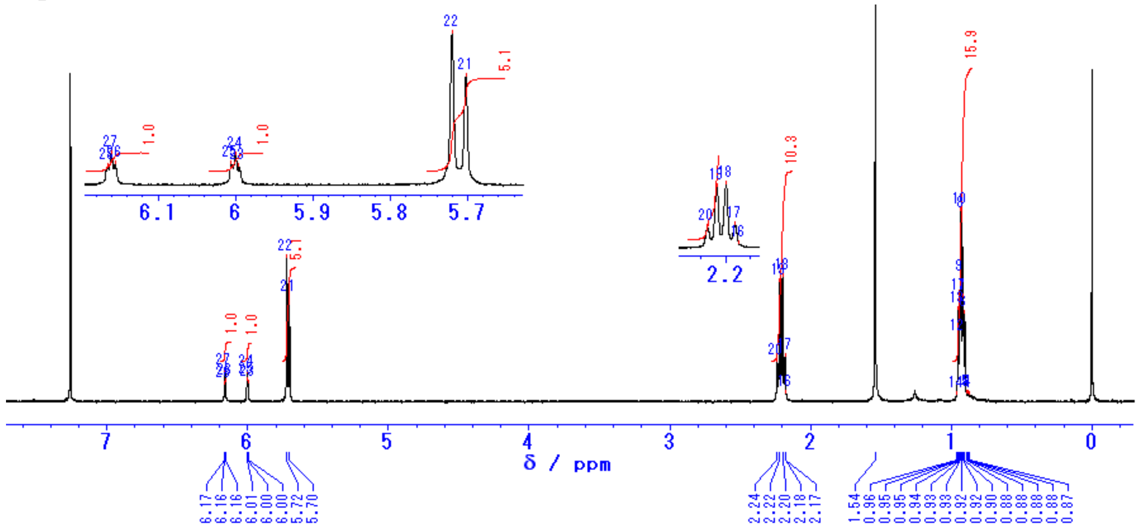

Figure S1a. ^{1}H-NMR spectrum of DEHPB in $CDCl_3$

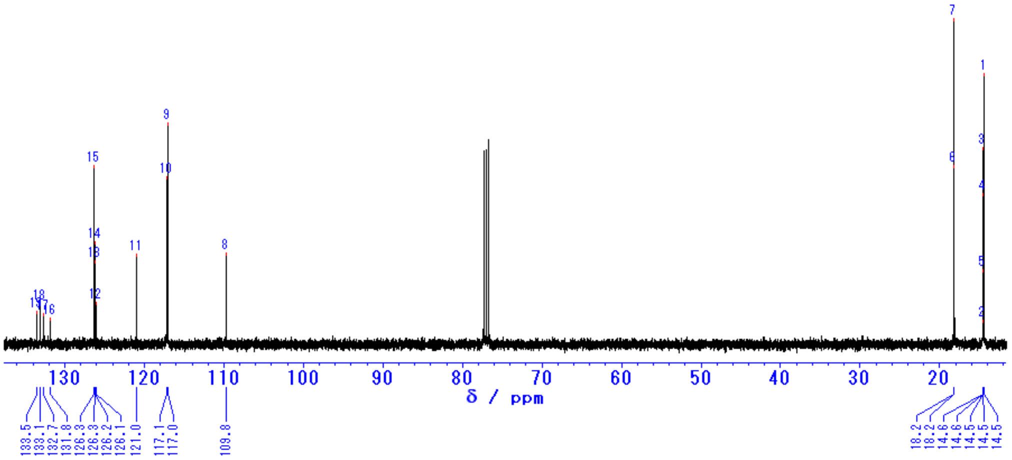

Figure S1b. ^{13}C-NMR spectrum of DEHPB in $CDCl_3$

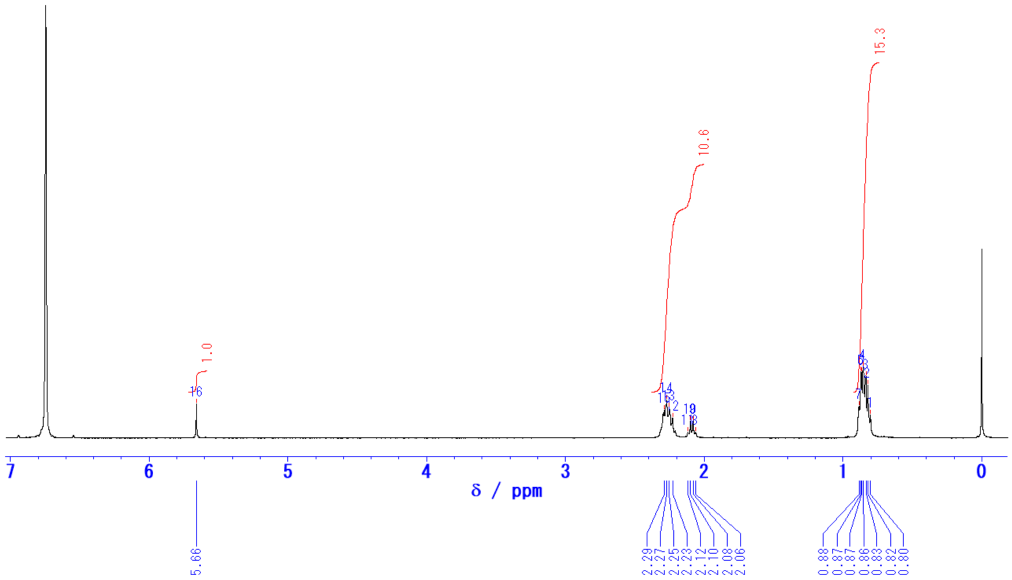

Figure S1c. ^{1}H-NMR spectrum of DEHPHAC **1a** in C_6D_6

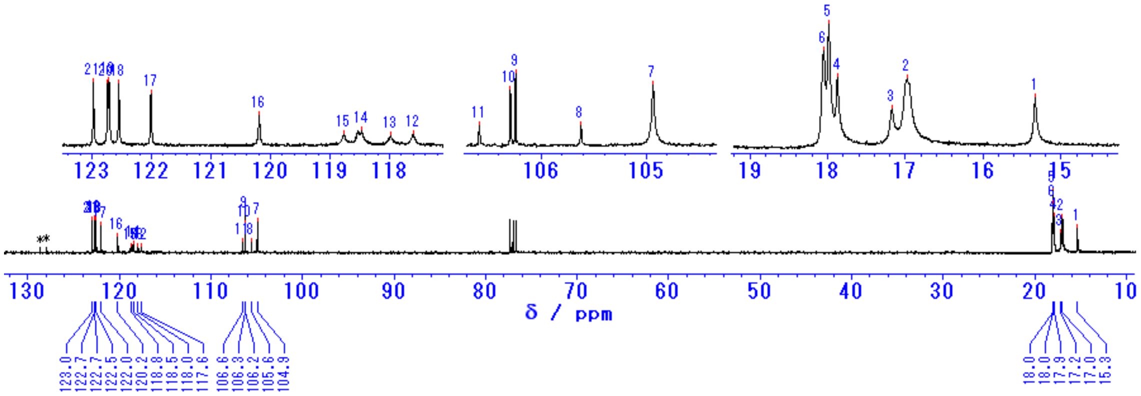

Figure 1d. ^{13}C-NMR spectrum of DEHPHAC **1a** in $CDCl_3$ and CS_2

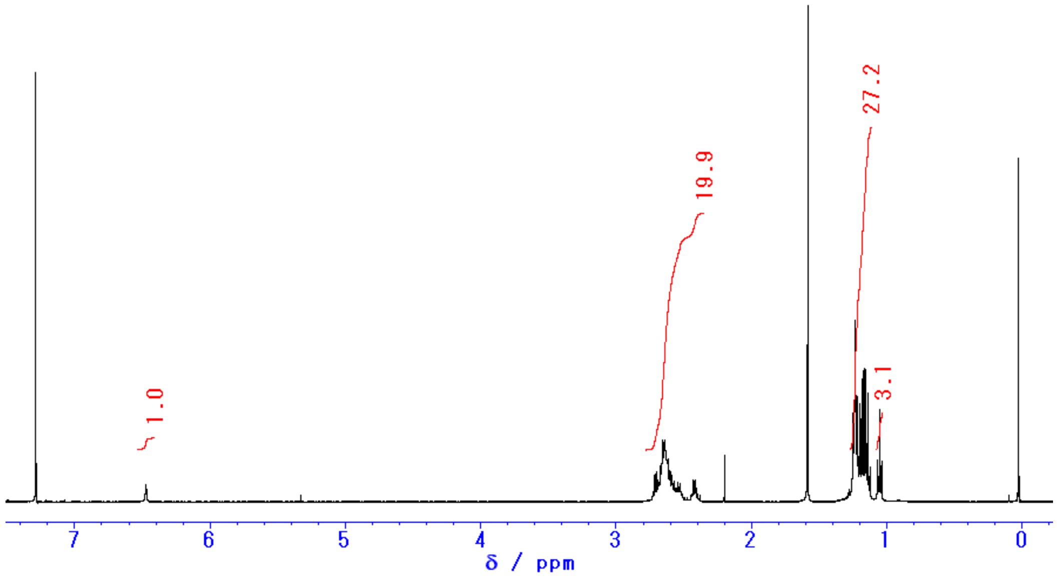

Figure S1e. ^{1}H-NMR spectrum of **2a** in $CDCl_3$.

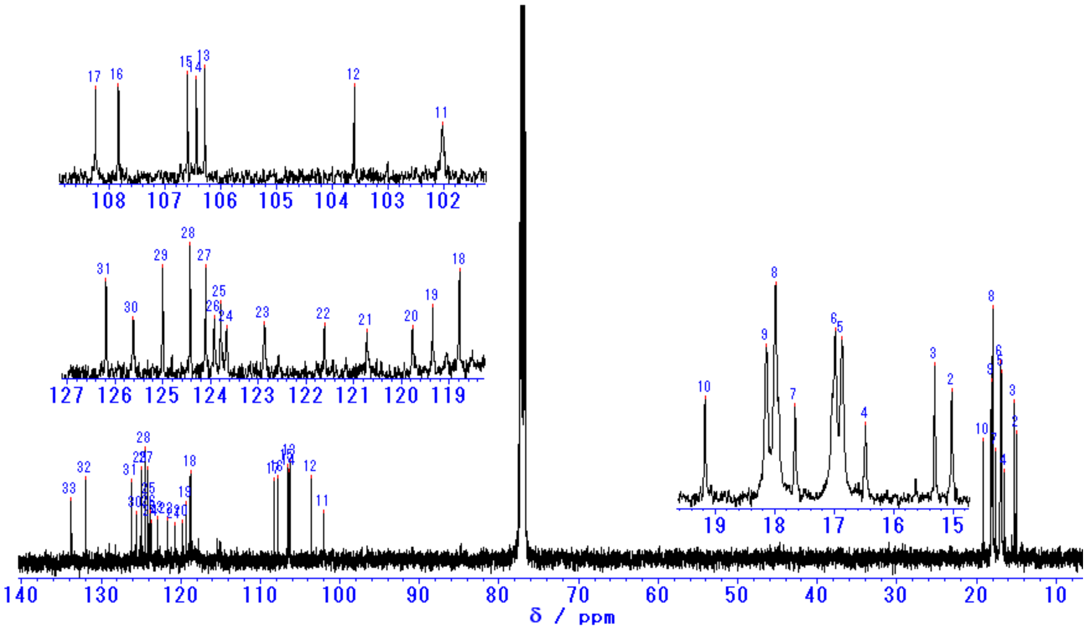

Figure S1f. ^{13}C-NMR spectrum of **2a** in $CDCl_3$ (The signals of central benzene moiety were not observed.).

2. Mass spectra

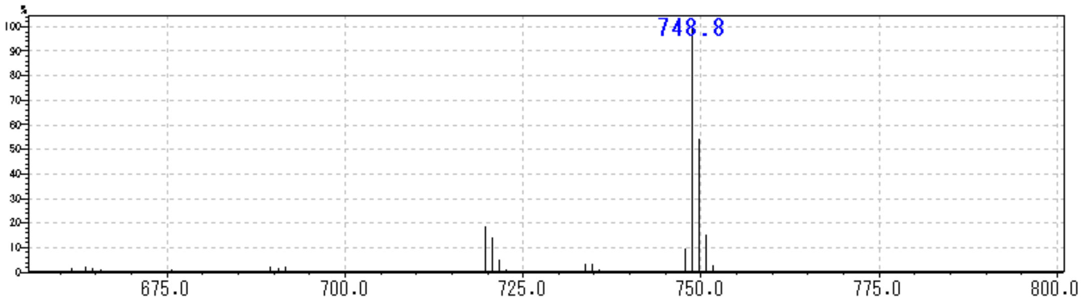

Figure S2a. DI-MS of DEHPB (Exact mass: 748.5192)

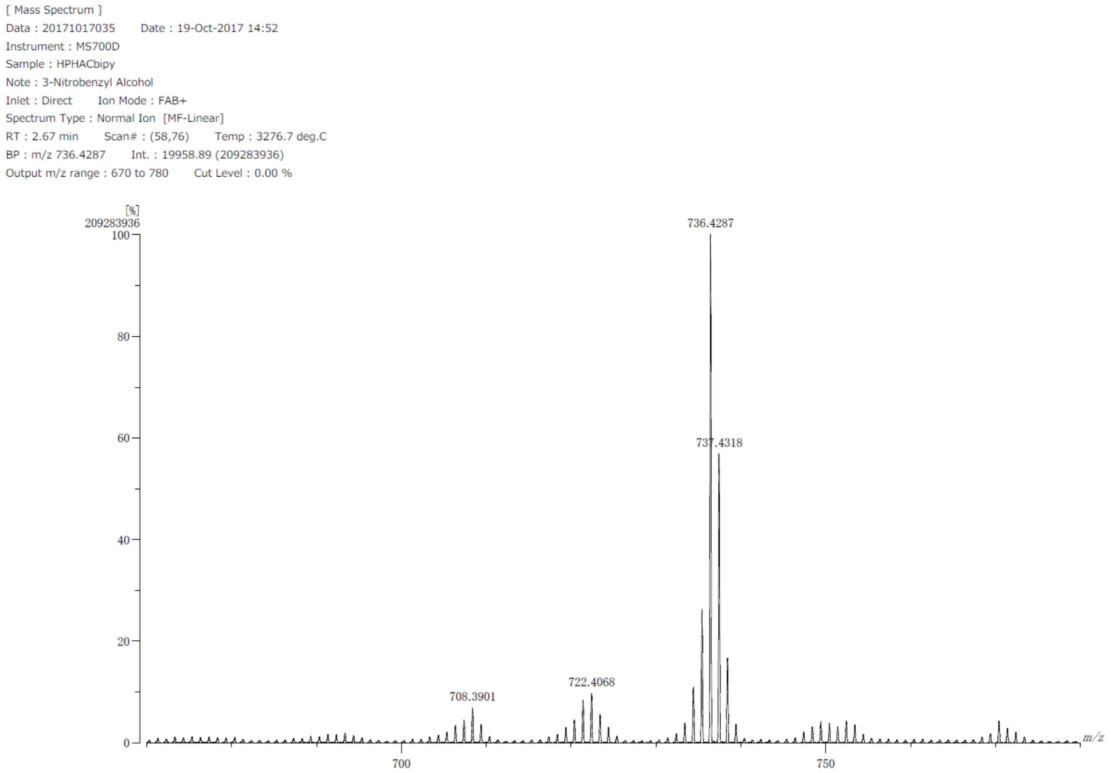

Figure S2b. HR-LDI-TOF MS of DEHPHAC **1a** (Exact mass: 736.4253)

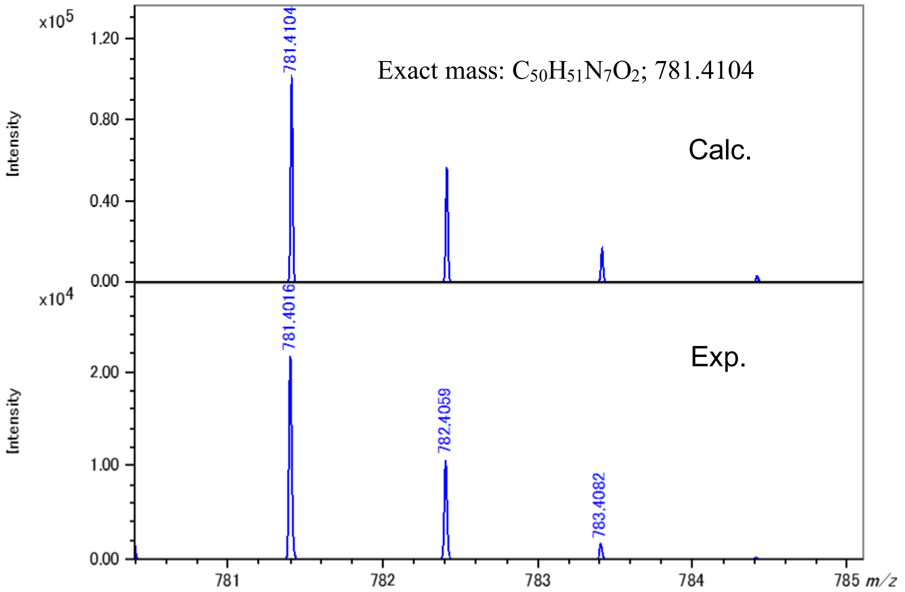

Figure S2c. High-resolution LDI-TOF mass spectrum of **2a** (Top: simulated, Bottom: observed).

3. IR spectra

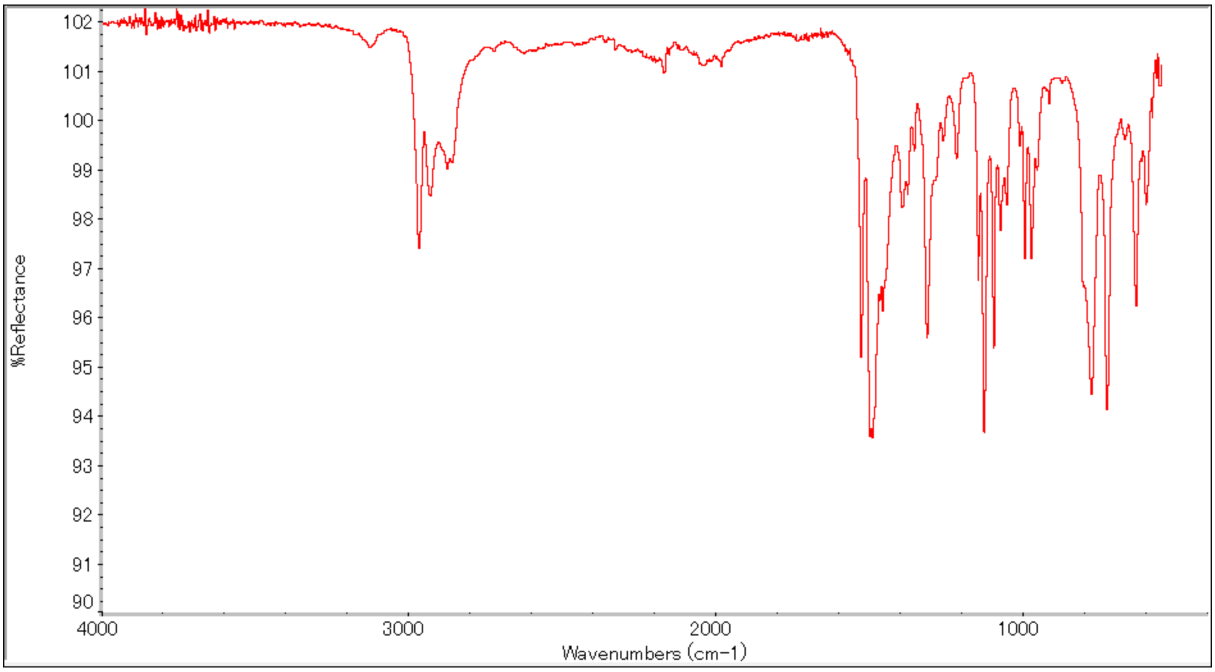

Figure S3a. IR spectrum of DEHPB

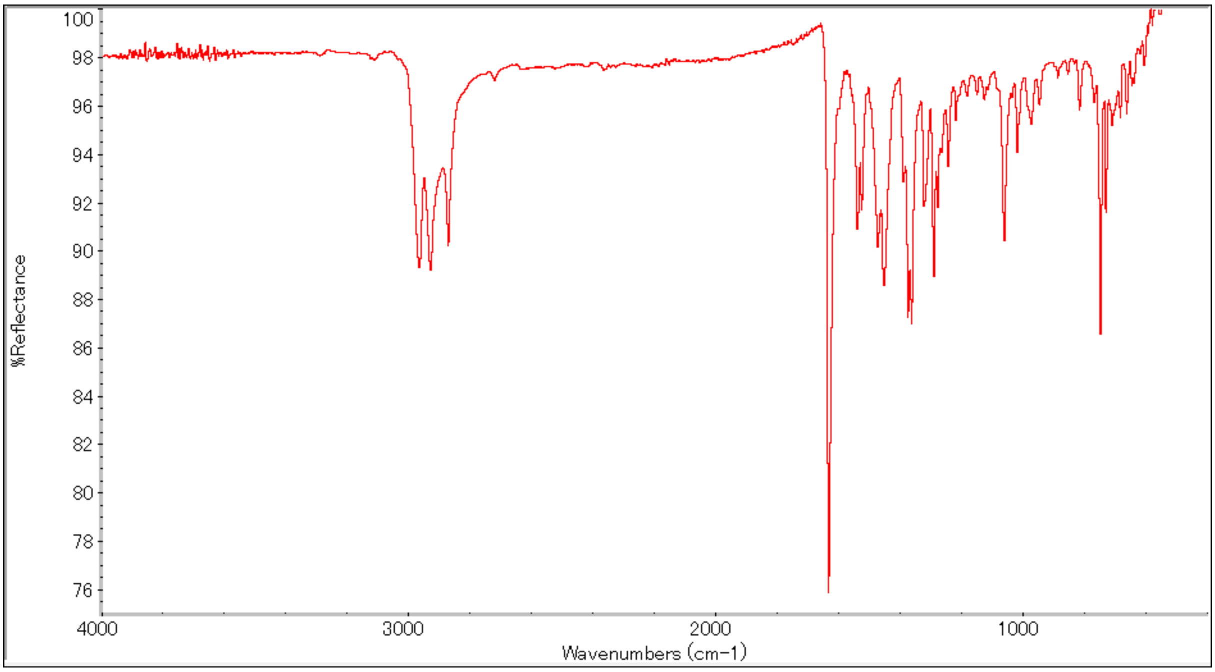

Figure S3b. IR spectrum of DEHPHAC **1a**

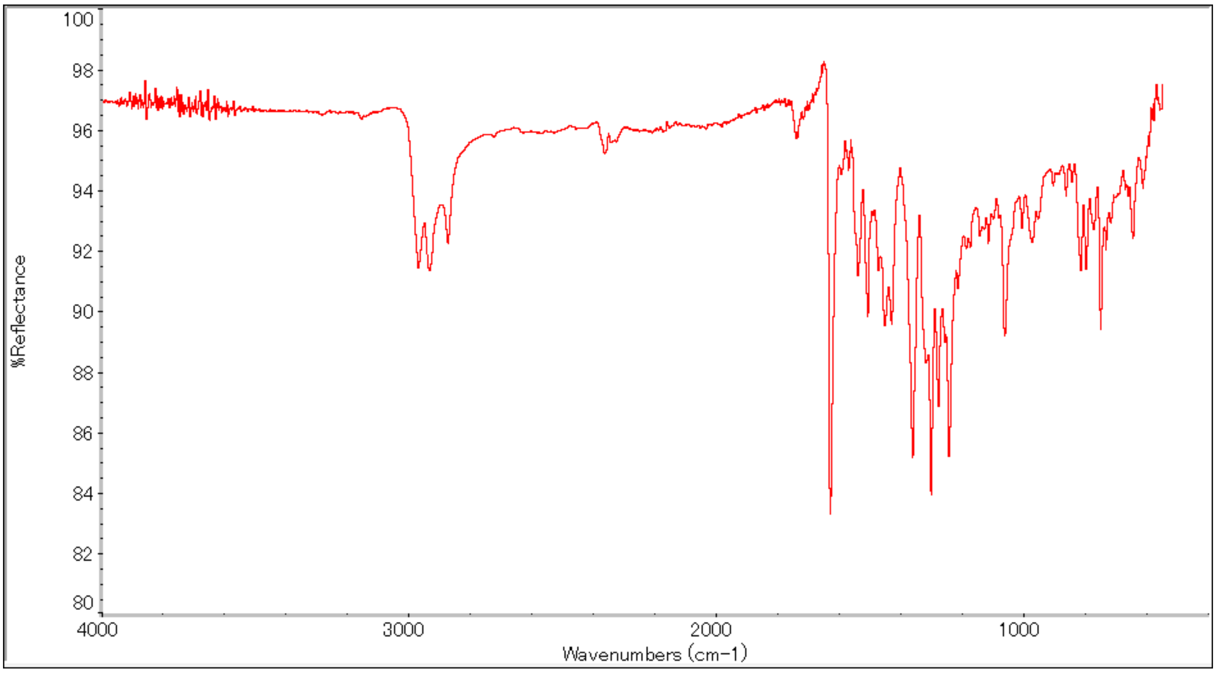

Figure S3c. IR spectrum of **2a**

4. Solvatochromism and dipole moments

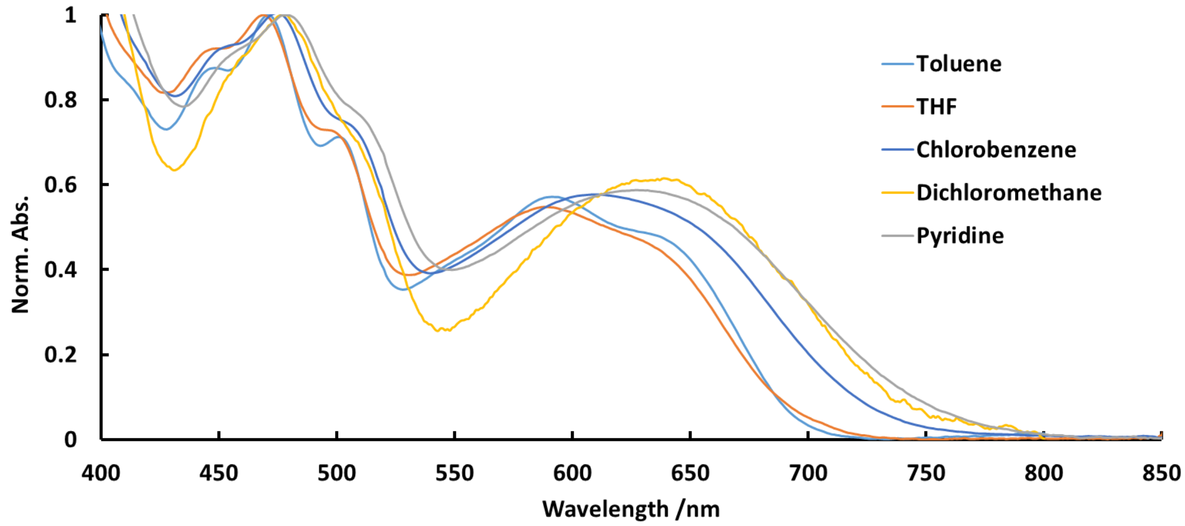

Figure S4a. Absorption spectra of **2a** in various solvents.

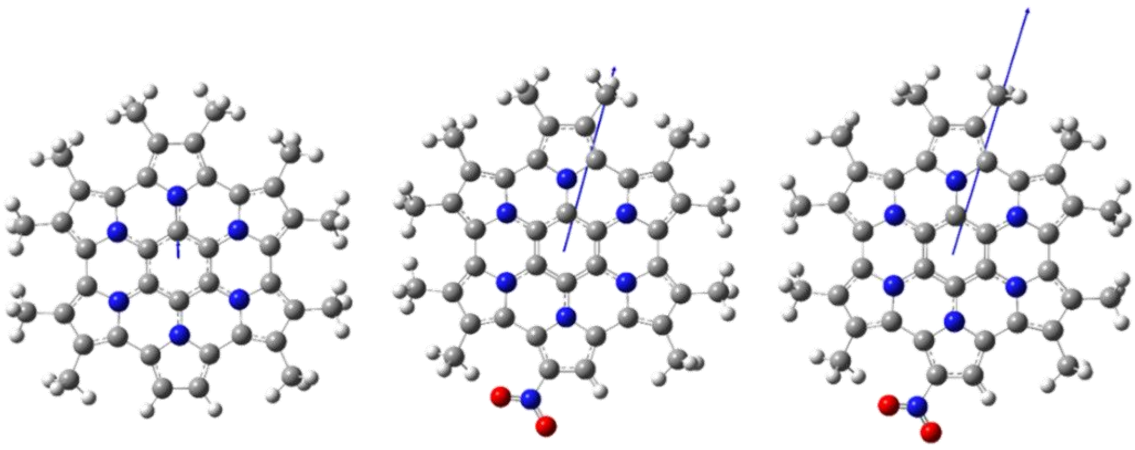

1b μ = 0.85 D

2b μ = 7.67 D

2b^{2+} μ = 10.13 D

Figure S4b. Dipole moments of **1b**, **2b** and **2b^{2+}** (Calculated by B3LYP/6-31G(d,p))

5. X-ray crystal structures[S1]

X-ray diffraction data were taken on Rigaku Varimax with Saturn724 diffractometer using multilayer mirror monochromated Mo Kα radiation (λ = 0.71073 Å) at 100±1 K. The crystals were mounted in cryoloops. Data collection was performed using CrystalClear software. Data reduction was performed using CrysAlisPro software. The data were corrected for Lorentz polarization and absorption effects. Structures were solved by using the processed data with Shelxt. All calculations were performed using the Rigaku CrystalStructure crystallographic software package.

Neutral

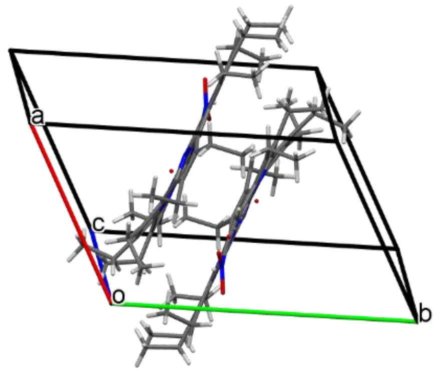

Figure S5a. Packing structure of **2a**

Table S4a. Crystallographic refinement data for **2a**

Formula	$C_{50}H_{51}N_7O_2$
Formula weight	782.00
Size	0.250×0.030×0.020 mm
Radiation	Mo*K*α
Temperature	100 K
Crystal system	triclinic
Space group	$P\bar{1}$ (#2)
Unit cell dimensions	a = 9.5224(4) Å
	b = 14.7625(5) Å
	c = 15.9432(6) Å
	α = 112.352(3) °
	β = 96.259(3) °
	γ = 108.189(3) °
Volume	1901.95(15) Å
Z	2
Density (calculated)	1.365 g·cm^{-3}
Absorption coefficient	0.085 mm^{-1}
F(000)	832.00
Reflections collected	31486
Independent reflections	8725
R_{int}	0.0449
R_1 [I>2sigma(I)]	0.0530

wR_2 (All reflections)	0.1412
GOF	1.026
CCDC No.	1989640

Radical cation

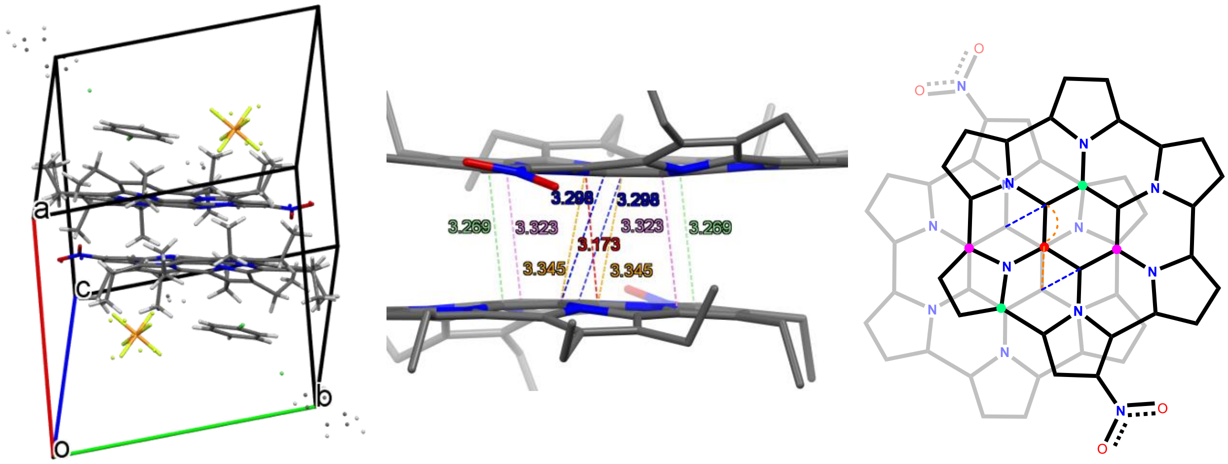

Figure S5b. Packing structure of **2a**$^{\cdot+}$ and interatomic distances (< 3.4 Å) in the π-dimeric structure.

Table S5b. Crystallographic refinement data for **2a**$^{\cdot+}$

Formula	$2C_{50}H_{51}N_7O_2 \cdot 3C_6H_5Cl \cdot 2PF_6$
Formula weight	2191.60
Size	0.120×0.060×0.050 mm
Radiation	Mo*K*α
Temperature	100 K
Crystal system	triclinic
Space group	$P\bar{1}$ (#2)
Unit cell dimensions	a = 12.8159(5) Å
	b = 13.8592(5) Å
	c = 15.2281(5) Å
	α = 79.519(3) °
	β = 71.137(3) °
	γ = 84.007(3) °
Volume	2513.80(16) Å
Z	1
Density (calculated)	1.448 $g \cdot cm^{-3}$
Absorption coefficient	0.211 mm^{-1}
F(000)	1144.00
Reflections collected	41723
Independent reflections	11525
R_{int}	0.0439
R_1 [I>2sigma(I)]	0.0698
wR_2 (All reflections)	0.1767
GOF	1.024
CCDC No.	1989647

Dication

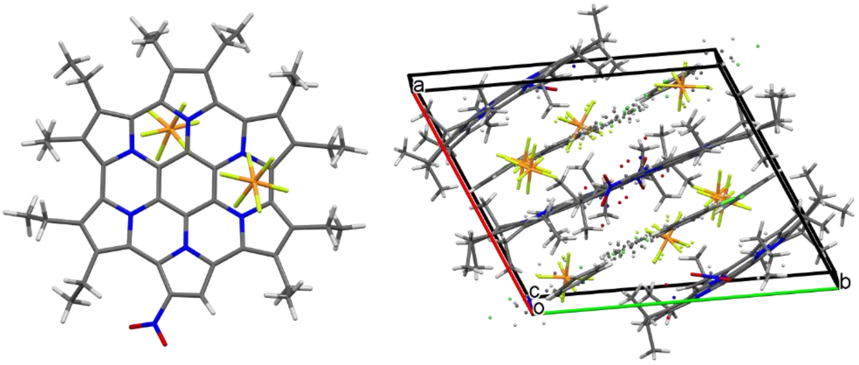

Figure S5c. Molecular structure and packing structure of **2a**$^{2+}$

Table S5c. Crystallographic refinement data for **2a**$^{2+}$

Formula	$2C_{50}H_{51}N_7O_2 \cdot 6(C_6H_5Cl) \cdot 4(PF_6)$
Formula weight	2819.20
Size	0.120×0.030×0.020 mm
Radiation	Mo*K*α
Temperature	100 K
Crystal system	triclinic
Space group	$P\bar{1}$ (#2)
Unit cell dimensions	a = 14.4794(4) Å
	b = 17.5827(4) Å
	c = 26.8407(7) Å
	α = 92.852(2) °
	β = 92.808(2) °
	γ = 113.182(2) °
Volume	6256.3(3) Å^3
Z	2
Density (calculated)	1.496 g·cm^{-3}
Absorption coefficient	0.287 mm^{-1}
F(000)	2912.00
Reflections collected	103951
Independent reflections	28714
R_{int}	0.0701
R_1 [I>2sigma(I)]	0.0931
wR_2 (All reflections)	0.2492
GOF	1.048
CCDC No.	1989648

6. Comparison of the ^{1}H-NMR spectra of 2a, 2a^{2+} and 1a^{2+}

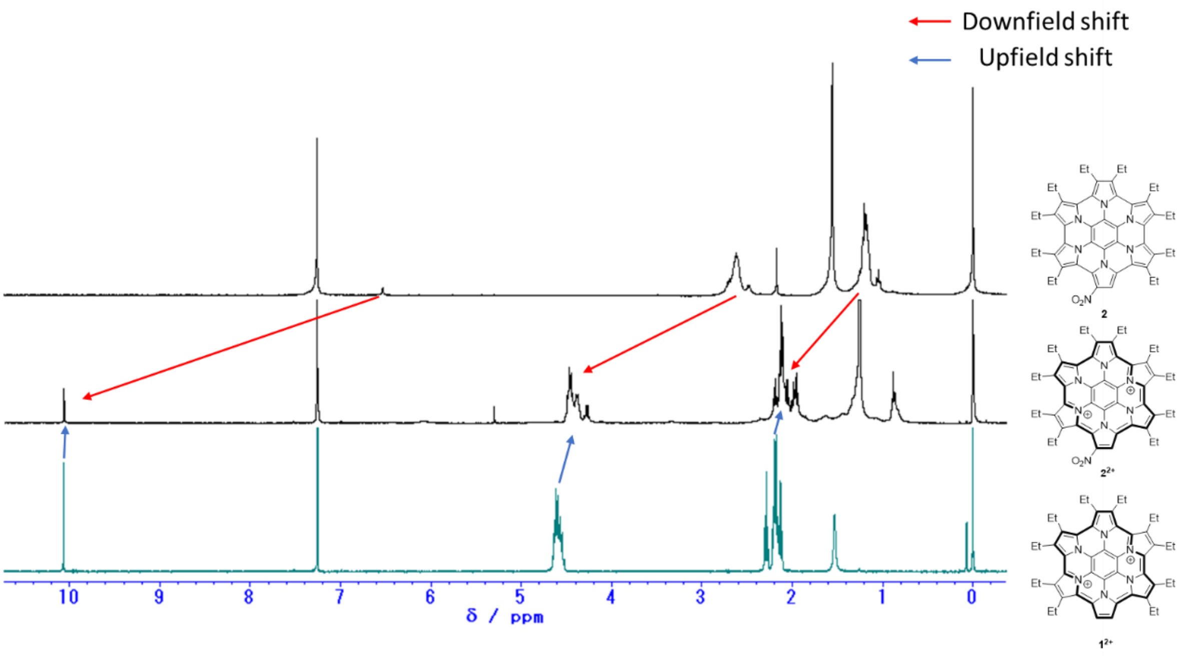

Figure S6. ^{1}H-NMR spectra of **2a** (top), **2a^{2+}** (middle), **1a^{2+}** (bottom) in $CDCl_3$.

7. TD-DFT calculations

The GAUSSIAN 09[S2] series of programs was used for all calculations. All molecules were fully optimized using the hybrid density functional at B3LYP level of theory with the 6-31G(d,p) basis set. Frequency calculations were conducted to ensure that these structures were indeed local minima. TD-DFT calculations were performed at the B3LYP/6-31+G(d,p) level of theory under vacuum.

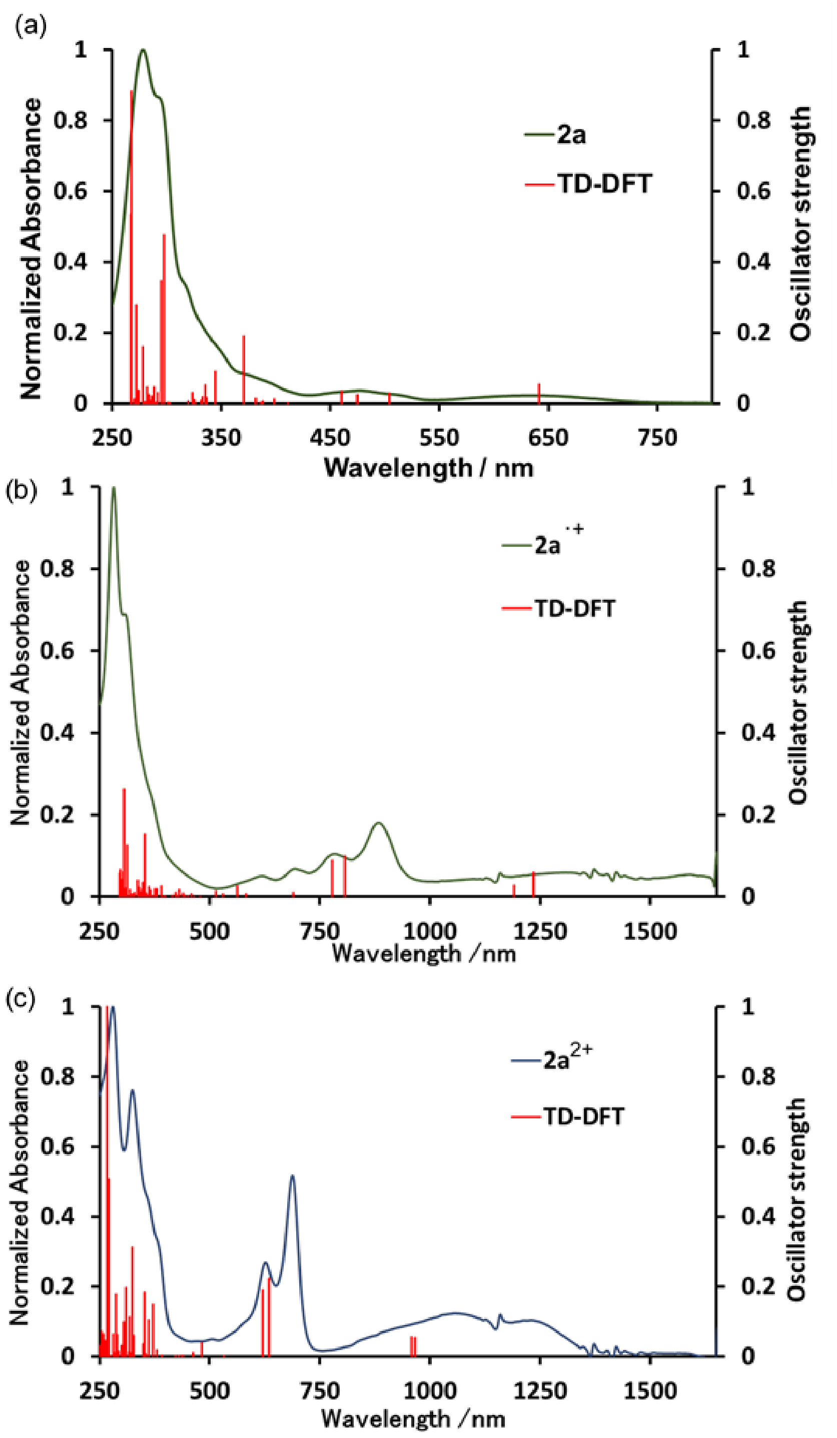

Figure S7. UV/Vis/NIR spectra and TD-DFT results of (a) **2a** and **2b**, (b) **2a**$^{\bullet+}$ and **2b**$^{\bullet+}$, and (c) **2b**$^{2+}$ and **2b**$^{2+}$, respectively. The calculations were performed at the B3LYP/6-31G+(d,p)//B3LYP/6-31G(d,p) level of theory.

Table S7. TD-DFT results of (a) **2b**, (b) **2b**$^{\bullet+}$ and (c) **2b**$^{2+}$.

(a) **2b** (HOMO = 168 and LUMO = 169)

```
Excited State   1:      Singlet-A      1.9325 eV  641.58 nm  f=0.0543  <S**2>=0.000
      168 -> 169         0.68457
      168 -> 170        -0.14810
 This state for optimization and/or second-order correction.
 Total Energy, E(TD-HF/TD-KS) =  -2076.62704338
 Copying the excited state density for this state as the 1-particle RhoCI density.
 Excited State   2:      Singlet-A      2.4595 eV  504.10 nm  f=0.0277  <S**2>=0.000
      168 -> 169         0.14244
      168 -> 170         0.68148
 Excited State   3:      Singlet-A      2.6104 eV  474.96 nm  f=0.0232  <S**2>=0.000
      166 -> 169         0.11468
      167 -> 169         0.68293
 Excited State   4:      Singlet-A      2.6934 eV  460.33 nm  f=0.0342  <S**2>=0.000
      166 -> 169         0.68440
      167 -> 169        -0.11295
 Excited State   5:      Singlet-A      3.0166 eV  411.01 nm  f=0.0004  <S**2>=0.000
      168 -> 171         0.69286
 Excited State   6:      Singlet-A      3.1080 eV  398.92 nm  f=0.0113  <S**2>=0.000
      165 -> 169         0.25182
      168 -> 172         0.64242
 Excited State   7:      Singlet-A      3.1943 eV  388.14 nm  f=0.0066  <S**2>=0.000
      165 -> 169         0.55415
      166 -> 170        -0.17238
      167 -> 170        -0.31406
      168 -> 172        -0.19785
 Excited State   8:      Singlet-A      3.2083 eV  386.45 nm  f=0.0011  <S**2>=0.000
      164 -> 169        -0.17442
      165 -> 169         0.14523
      166 -> 170         0.60930
      168 -> 172        -0.14883
      168 -> 173        -0.15823
 Excited State   9:      Singlet-A      3.2458 eV  381.99 nm  f=0.0144  <S**2>=0.000
      164 -> 169         0.29930
      165 -> 169         0.26755
      166 -> 171        -0.11337
      167 -> 170         0.53437
 Excited State  10:      Singlet-A      3.3425 eV  370.93 nm  f=0.1901  <S**2>=0.000
      164 -> 169         0.58094
      165 -> 169        -0.12874
      166 -> 170         0.19736
      166 -> 171         0.11391
      167 -> 170        -0.24139
```

167 -> 171 0.10893

(b) **2b**$^{\cdot+}$ (SOMO = 168A)

Excited State 1: 2.003-A 1.0045 eV 1234.30 nm f=0.0586 <S**2>=0.753
164B -> 168B -0.19050
167B -> 168B 0.97133
This state for optimization and/or second-order correction.
Total Energy, E(TD-HF/TD-KS) = -2076.45557973
Copying the excited state density for this state as the 1-particle RhoCI density.
Excited State 2: 2.009-A 1.0406 eV 1191.50 nm f=0.0279 <S**2>=0.759
165B -> 168B -0.25309
166B -> 168B 0.95850
Excited State 3: 2.031-A 1.5366 eV 806.86 nm f=0.0982 <S**2>=0.781
164B -> 168B 0.20154
165B -> 168B 0.92782
166B -> 168B 0.22163
Excited State 4: 2.040-A 1.5923 eV 778.64 nm f=0.0882 <S**2>=0.791
164B -> 168B 0.94156
165B -> 168B -0.19514
167B -> 168B 0.16601
Excited State 5: 2.119-A 1.7981 eV 689.53 nm f=0.0102 <S**2>=0.872
168A -> 169A 0.90799
168A -> 170A -0.32243
163B -> 168B -0.12260
Excited State 6: 2.090-A 2.1284 eV 582.53 nm f=0.0064 <S**2>=0.842
168A -> 169A 0.18880
168A -> 170A 0.19058
163B -> 168B 0.93694
Excited State 7: 2.526-A 2.2031 eV 562.78 nm f=0.0260 <S**2>=1.345
165A -> 169A 0.11231
166A -> 169A 0.23063
167A -> 169A -0.11768
167A -> 170A 0.10107
168A -> 169A 0.17619
168A -> 170A 0.76710
163B -> 168B -0.26788
166B -> 169B -0.30904
167B -> 169B 0.21715
167B -> 170B -0.15128
Excited State 8: 3.276-A 2.3345 eV 531.10 nm f=0.0061 <S**2>=2.434
166A -> 169A -0.10107
167A -> 169A -0.42876
167A -> 170A 0.21779
168A -> 169A -0.12726

168A -> 170A -0.21147
168A -> 172A 0.10525
168A -> 173A 0.13210
166B -> 169B 0.22036
167B -> 169B 0.68905
167B -> 170B -0.26284
Excited State 9: 3.122-A 2.4079 eV 514.91 nm f=0.0132 <S**2>=2.187
166A -> 169A -0.43133
166A -> 170A 0.18222
168A -> 169A 0.18632
168A -> 170A 0.37315
168A -> 172A -0.15041
168A -> 173A 0.14023
166B -> 169B 0.61811
166B -> 170B -0.23848
167B -> 169B -0.16671
Excited State 10: 2.061-A 2.5868 eV 479.30 nm f=0.0003 <S**2>=0.812
161B -> 168B -0.54771
162B -> 168B 0.81277

(c) **2b**$^{2+}$ (HOMO = 167, LUMO = 168)
Excited State 1: Singlet-A 1.2821 eV 967.03 nm f=0.0529 <S**2>=0.000
164 -> 168 -0.19150
165 -> 168 -0.20105
166 -> 168 0.45385
167 -> 168 0.46057
This state for optimization and/or second-order correction.
Total Energy, E(TD-HF/TD-KS) = -2076.13567202
Copying the excited state density for this state as the 1-particle RhoCI density.
Excited State 2: Singlet-A 1.2935 eV 958.53 nm f=0.0551 <S**2>=0.000
164 -> 168 -0.17771
165 -> 168 0.22126
166 -> 168 -0.44728
167 -> 168 0.46229
Excited State 3: Singlet-A 1.9528 eV 634.91 nm f=0.2236 <S**2>=0.000
164 -> 169 0.10618
165 -> 168 0.61509
166 -> 168 0.26514
167 -> 169 0.15811
Excited State 4: Singlet-A 1.9992 eV 620.16 nm f=0.1907 <S**2>=0.000
164 -> 168 0.63781
165 -> 169 -0.10379
166 -> 169 -0.10076
167 -> 168 0.23395

Excited State 5: Singlet-A 2.3287 eV 532.43 nm f=0.0005 <S**2>=0.000
163 -> 168 0.70450
Excited State 6: Singlet-A 2.5676 eV 482.88 nm f=0.0399 <S**2>=0.000
164 -> 169 -0.10230
167 -> 169 0.66722
Excited State 7: Singlet-A 2.6776 eV 463.04 nm f=0.0101 <S**2>=0.000
166 -> 169 0.67331
Excited State 8: Singlet-A 2.8232 eV 439.16 nm f=0.0017 <S**2>=0.000
161 -> 168 -0.31421
162 -> 168 0.62447
Excited State 9: Singlet-A 2.8849 eV 429.77 nm f=0.0004 <S**2>=0.000
161 -> 168 0.62214
162 -> 168 0.30353
Excited State 10: Singlet-A 2.9377 eV 422.05 nm f=0.0011 <S**2>=0.000
160 -> 168 0.68458

8. ACID plots and NICS[S3] calculations

ACID plots (CSGT/B3LYP/6-31G+(d,p)) of **2b** and **2b**$^{2+}$ were calculated by using the method developed by Herges based on the optimized ground-state geometries.[S4] For ACID calculations, the magnetic field is perpendicular to the molecular center and points out through the paper. Blue and red arrows indicate paratropic (counterclockwise) and diatropic (clockwise) ring current, respectively. (Isovalue: 0.03)

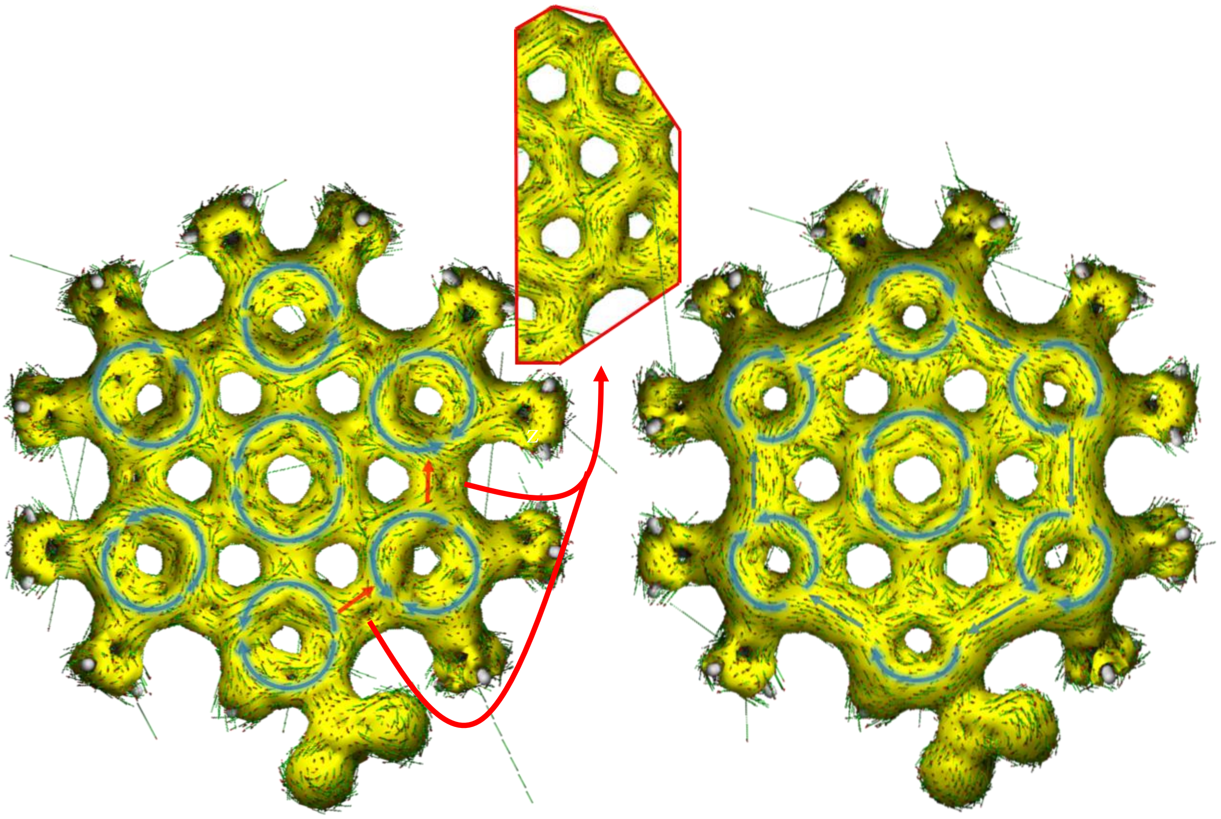

Figure S8a. ACID plots of **2b** (left) and **2b**$^{2+}$ (right).

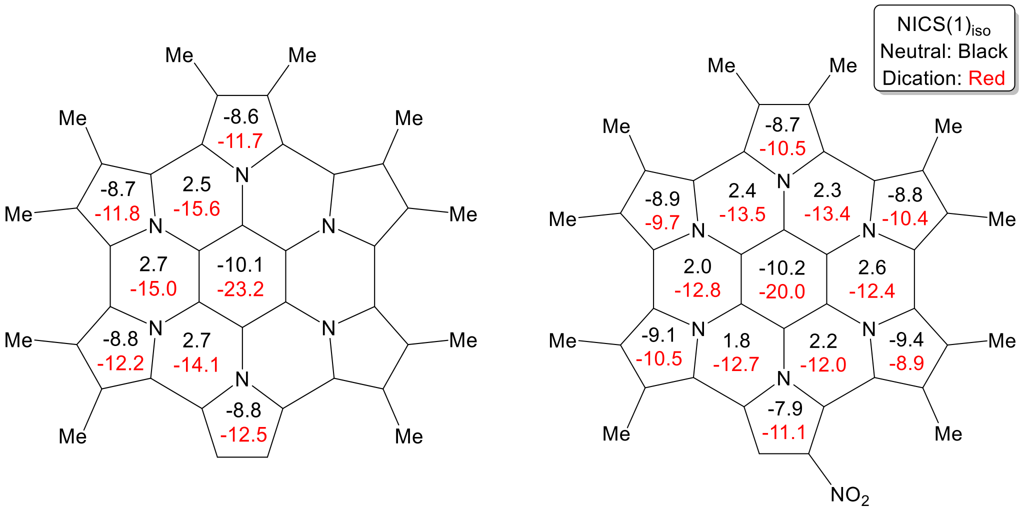

Figure S8b. NICS values of **1b**/**1b**$^{2+}$ (left) and **2b**/**2b**$^{2+}$ (right) (Calculated by HF/6-311G+(d,p)//B3LYP/6-31G(d,p)).

9. Possible resonance effect

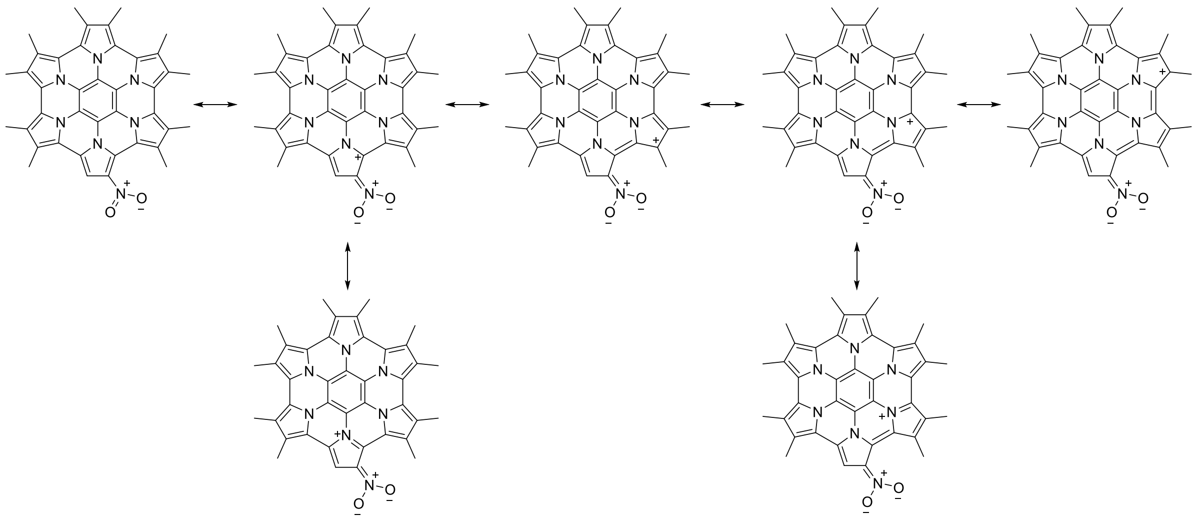

Scheme S9. Possible resonance structures of **2b**.

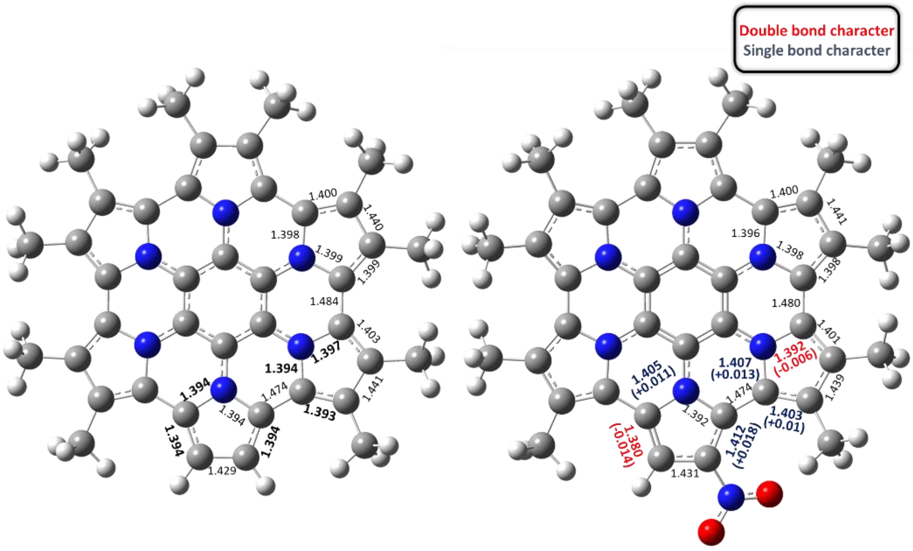

Figure S9a. Bond lengths of **1b** (left) and **2b** (right) in the optimized structures.

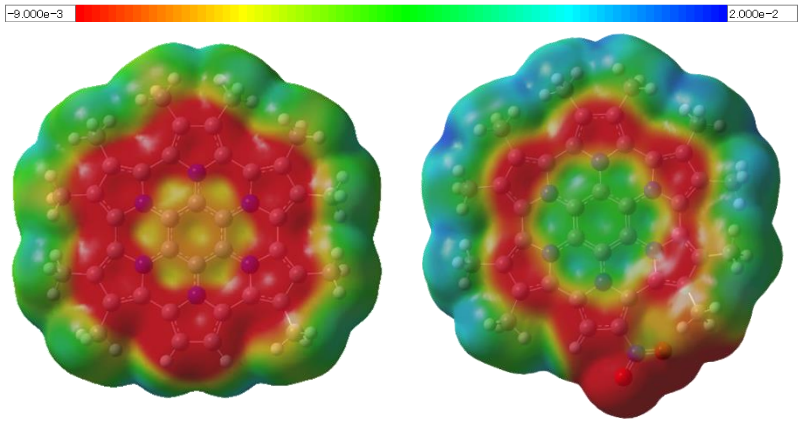

Figure S9b. ESP surface of **1b** (left) and **2b** (right).

10. Atomic coordinates of the optimized structures

The calculations were performed at the R(U)B3LYP/6-31G(d,p) level of theory.

・Neutral **2b**

N -2.26180300 -1.33292800 -0.05727000
N 0.23377000 2.70937600 -0.04137700
N -2.18470000 1.40309800 0.01913600
N 2.56539900 1.27328700 0.02035000
C -1.02007600 0.69099500 0.00713200
C -1.05433600 -0.68424600 -0.04337100
C -3.44809300 0.80373300 -0.13724000
C -2.21270100 2.78779500 -0.11858500
C 0.19388600 1.34538000 -0.02365800
C 3.79474100 0.60635700 0.02794900
C -0.91946800 3.49729000 0.00536400
C 1.41612300 3.43941600 0.09214900
C -3.48297200 -0.66557800 -0.02301000
C -4.39828800 -1.73371300 0.09991900
C -3.54966200 3.09089300 -0.40600300
C 4.74396400 1.63072600 0.09636100
C 0.99256200 4.76558900 0.24529900
C -0.44758500 4.79934000 0.19749100
C -4.31174500 1.87100800 -0.42490300
C 1.36702700 0.62316600 0.00632200
N 2.49290100 -1.47197200 0.05487400
N 0.08643400 -2.77225600 -0.04172300
C 0.11928500 -1.40855200 -0.02901900
C -1.10011100 -3.49797800 0.02275000
C 1.22872000 -3.57385200 0.00492500
C 1.33058900 -0.75489600 0.02622700
C 2.68080200 2.66638400 0.06712600
C 2.52846800 -2.86659000 -0.05360300
C 3.75228200 -0.87317700 -0.04230500
C -2.35713300 -2.73282100 0.01964900
C -3.71057500 -2.98869100 0.10320300
H -4.20507600 -3.94114300 0.19582400
C 0.73433600 -4.88125600 0.11180200
C 4.05950400 2.89854100 0.09168400
C 4.63599100 -1.94265300 -0.22651800
C 3.88107700 -3.17038400 -0.23315100
C -0.70469800 -4.83079500 0.10842600
N -5.79877600 -1.65916600 0.35113400
O -6.44923300 -2.70915300 0.26897200
O -6.28305100 -0.56706000 0.67654200
C -1.27142700 6.04016500 0.38597900
H -1.39636300 6.61075600 -0.54321300
H -2.26588400 5.80286600 0.76639700
H -0.80304000 6.71373000 1.10993400
C 1.85251400 5.97906600 0.45050200
H 2.72196000 5.75900400 1.07295300
H 2.21886900 6.39722900 -0.49587500
H 1.29190800 6.77419000 0.94863600
C -4.11514700 4.44804200 -0.70740300
H -4.51834900 4.94305200 0.18517100
H -3.36016800 5.10638400 -1.14106200
H -4.93663100 4.37817700 -1.42565400
C -5.76650900 1.80924500 -0.78278700
H -6.02608500 0.86187800 -1.25320100
H -6.41325000 1.91117000 0.09426300
H -6.01986700 2.61328100 -1.47980500
C -1.60862400 -6.02500300 0.19757500
H -1.51379000 -6.53603900 1.16369400
H -2.65686100 -5.74292900 0.07981000
H -1.37915600 -6.76266200 -0.57958400
C 1.51112500 -6.15905700 0.24647600
H 1.76650700 -6.60266200 -0.72469800
H 2.44236200 -6.01053900 0.79698200
H 0.92660700 -6.90603300 0.79146000
C 4.48036000 -4.53110900 -0.43931900
H 4.82215200 -4.98679900 0.49901600
H 3.76661300 -5.21592500 -0.89929500
H 5.34989600 -4.48116600 -1.10103500
C 6.12223500 -1.86991800 -0.42474100
H 6.41222900 -0.96383400 -0.95940700
H 6.67488500 -1.88854600 0.52347900
H 6.47939800 -2.71934200 -1.01325300
C 6.23688800 1.49672600 0.19693200
H 6.52224800 0.57680900 0.70945000
H 6.73073400 1.49661300 -0.78375000
H 6.66196500 2.32718700 0.76759500
C 4.77115200 4.22159400 0.09305400
H 4.21464700 4.97761500 -0.46282800

H 4.93747500 4.61096900 1.10603900
H 5.75285400 4.13324100 -0.38009000

·Radical cation **2b**$^{\cdot+}$

N -2.23586900 -1.37141000 -0.11592200
N 0.18260600 2.70418400 -0.06195200
N -2.20386700 1.36021400 -0.03060400
N 2.53473300 1.31078600 -0.03224600
C -1.03288100 0.66930700 -0.07485900
C -1.04386700 -0.70308600 -0.13425900
C -3.45942200 0.74589800 -0.12535400
C -2.25522300 2.75303600 -0.11130100
C 0.16684300 1.34339100 -0.09221300
C 3.76981700 0.66107600 -0.00133000
C -0.97778800 3.47079100 -0.01747000
C 1.36137100 3.44753700 0.04968800
C -3.46712700 -0.72191600 -0.03802600
C -4.35727000 -1.81415000 0.10692700
C -3.62322100 3.04217800 -0.30876400
C 4.71238600 1.72039300 0.08800900
C 0.91216100 4.78655700 0.18469300
C -0.51213100 4.79908000 0.15156400
C -4.36073700 1.81729300 -0.32718300
C 1.34913500 0.64327800 -0.08105500
N 2.50709500 -1.42557800 -0.03974600
N 0.13395700 -2.76538100 -0.10817300
C 0.13979700 -1.40492400 -0.12768200
C -1.03630000 -3.50831000 -0.04469300
C 1.29526500 -3.54300400 -0.05161500
C 1.33636300 -0.73084100 -0.08708500
C 2.61973700 2.70241600 0.03322000
C 2.56884100 -2.81621800 -0.07167100
C 3.75797400 -0.80000000 -0.04750900
C -2.29893600 -2.76891700 -0.03384200
C -3.65598100 -3.04713200 0.08997600
H -4.13125300 -4.00848400 0.19870100
C 0.81540700 -4.87203600 0.04544100
C 4.01544700 2.95935500 0.08567400
C 4.68119800 -1.87662500 -0.10798500
C 3.95711500 -3.10229000 -0.12264800
C -0.61268000 -4.84596400 0.03821300
N -5.76511300 -1.76670600 0.39217400
O -6.40150000 -2.81132700 0.25394300
O -6.23097300 -0.69803600 0.79554400
C -1.36053300 6.02481500 0.32042900
H -1.59563900 6.50024700 -0.63920600
H -2.30231100 5.79083000 0.81802300
H -0.85097300 6.77306900 0.93109400
C 1.76797400 6.00705800 0.35068800
H 2.58443400 5.83703500 1.05495700
H 2.20725000 6.32759400 -0.60119000
H 1.18349300 6.84755400 0.72761100
C -4.23097100 4.39753700 -0.51587200
H -4.54172500 4.85710000 0.43004800
H -3.53596400 5.07792900 -1.00917600
H -5.12272500 4.33281000 -1.14263400
C -5.83198000 1.73668700 -0.59891000
H -6.10029900 0.80601700 -1.09642300
H -6.41996900 1.78479800 0.32241800
H -6.14610800 2.56365800 -1.24008000
C -1.50583300 -6.04829300 0.12317800
H -1.49713400 -6.48783300 1.12750400
H -2.54039200 -5.79526100 -0.11632600
H -1.19136300 -6.82809400 -0.57677000
C 1.61709900 -6.13514300 0.16014000
H 1.99518700 -6.47248700 -0.81230300
H 2.47331700 -6.01412700 0.82656800
H 1.00242900 -6.94260900 0.56317800
C 4.57603100 -4.46649000 -0.19616800
H 4.67557400 -4.92365800 0.79539400
H 3.98396000 -5.14075600 -0.81680600
H 5.57705800 -4.42365800 -0.62906200
C 6.17635100 -1.77087400 -0.16467700
H 6.49756900 -0.94847700 -0.80573500
H 6.61334700 -1.61400600 0.82846100
H 6.61915200 -2.68449400 -0.56455700

C	6.20358700	1.59061600	0.19482900
H	6.48553800	0.76174000	0.84594800
H	6.67498000	1.42454700	-0.78102900
H	6.64392100	2.49477200	0.61789700
C	4.68175300	4.30377200	0.11841300
H	4.16780700	5.01903900	-0.52518900
H	4.71144700	4.72535200	1.13013100
H	5.71176100	4.23685400	-0.23552600

・Dication **2b**$^{2+}$

N	-2.27807700	-1.27232800	0.23457300
N	0.29599900	2.68457000	0.26475100
N	-2.14208300	1.45135400	0.27926100
N	2.58163400	1.19961900	0.26523000
C	-1.00635200	0.71427900	0.43263200
C	-1.06993500	-0.65764900	0.39890000
C	-3.39999800	0.88237100	0.07007500
C	-2.11659200	2.83711900	0.07294900
C	0.22256200	1.33440500	0.42373700
C	3.77730100	0.49517600	0.12102500
C	-0.82507900	3.49169500	0.12253800
C	1.51123900	3.37132300	0.15273000
C	-3.47804100	-0.56930300	0.13443500
C	-4.42198300	-1.63195600	0.01671900
C	-3.46589600	3.16956600	-0.25924900
C	4.77093200	1.52761500	-0.03437900
C	1.11905800	4.74638500	0.00826100
C	-0.28791500	4.81987900	-0.00236000
C	-4.24191700	1.98464200	-0.27910900
C	1.37512500	0.58501400	0.41852700
N	2.44070500	-1.52631300	0.25132800
N	0.01754100	-2.75876900	0.23312300
C	0.08179400	-1.40926400	0.39711200
C	-1.17980400	-3.44359100	0.10564500
C	1.14664500	-3.57451600	0.08785400
C	1.30675100	-0.78730600	0.41247200
C	2.71602200	2.58256000	0.14127900
C	2.43063500	-2.90586000	0.07933400
C	3.70512700	-0.94335400	0.09535800
C	-2.39667400	-2.65899100	0.11794500
C	-3.77938300	-2.87720800	-0.01651400
H	-4.29739800	-3.81929000	-0.10808500
C	0.60353000	-4.88858300	-0.08610900
C	4.13178800	2.77983700	-0.03818900
C	4.57707900	-2.06310300	-0.14037000
C	3.80895800	-3.24361600	-0.14880300
C	-0.80950100	-4.80157700	-0.08230800
N	-5.86748500	-1.53471800	0.06597800
O	-6.50113400	-2.47305200	-0.40228100
O	-6.33385500	-0.53900100	0.61718900
C	-1.07985300	6.08797000	-0.09962900
H	-1.37592500	6.30451800	-1.13223900
H	-1.98319300	6.04113800	0.50856700
H	-0.49774600	6.94067800	0.25170300
C	2.03796200	5.92301100	-0.11276800
H	2.83804700	5.88767500	0.62851000
H	2.49700300	5.97202700	-1.10613400
H	1.49915400	6.85889800	0.03279700
C	-3.99046000	4.53808200	-0.56970600
H	-4.06298500	5.15079900	0.33538600
H	-3.35402400	5.06312600	-1.28447400
H	-4.99098900	4.48933100	-0.99740300
C	-5.68282400	1.91836600	-0.67942900
H	-5.89190500	1.03222800	-1.27926900
H	-6.34175300	1.87722000	0.19225600
H	-5.96048000	2.78934300	-1.27337500
C	-1.75319700	-5.95354600	-0.25218300
H	-1.67033000	-6.65812700	0.58257000
H	-2.79171500	-5.62413600	-0.29883600
H	-1.54143000	-6.50828500	-1.17120500
C	1.34967600	-6.17709800	-0.25294100
H	1.75033800	-6.28508400	-1.26722200
H	2.18172900	-6.25993900	0.44837800
H	0.68795800	-7.02649500	-0.07726900
C	4.33511200	-4.62871800	-0.36752700
H	4.24367800	-5.23454200	0.53998800
H	3.79508100	-5.13931900	-1.16815300
H	5.38887800	-4.61874200	-0.64227700

C	6.05823900	-1.99651300	-0.35415400
H	6.31880400	-1.29017100	-1.14520500
H	6.58005000	-1.69111900	0.55860400
H	6.46068500	-2.96656600	-0.64233600
C	6.24946100	1.31850900	-0.15846100
H	6.60230800	0.55696700	0.53812400
H	6.53283300	1.00971300	-1.17067200
H	6.79526500	2.23463900	0.06565100
C	4.82194400	4.09789900	-0.21169200
H	4.37803500	4.67510500	-1.02583200
H	4.76739500	4.70290500	0.69895500
H	5.87570100	3.96431200	-0.45235900

11. References

[S1] (a) CrystalStructure: Reflection collection, data reduction, and crystal structure analysis packages, Rigaku (3-9-12 Akishima, Tokyo, Japan) and Rigaku/MSC (9009 New Trails Dr., The Woodlands, TX 77381 USA). (b) Sheldrick, G. M. *Acta. Cryst.* **2014**, *A70*, C1437. (c) SHELXL: Sheldrick, G. M. *Acta Crystallogr*. **2008**, *A78*, 112.

[S2] Gaussian 09, Revision D.01, Frisch, M. J.; Trucks, G. W.; Schlegel, H. B.; Scuseria, G. E.; Robb, M. A.; Cheeseman, J. R.; Scalmani, G.; Barone, V.; Mennucci, B.; Petersson, G. A.; Nakatsuji, H.; Caricato, M.; Li, X.; Hratchian, H. P.; Izmaylov, A. F.; Bloino, J.; Zheng, G.; Sonnenberg, J. L.; Hada, M.; Ehara, M.; Toyota, K.; Fukuda, R.; Hasegawa, J.; Ishida, M.; Nakajima, T.; Honda, Y.; Kitao, O.; Nakai, H.; Vreven, T.; Montgomery, Jr., J. A.; Peralta, J. E.; Ogliaro, F.; Bearpark, M.; Heyd, J. J.; Brothers, E.; Kudin, K. N.; Staroverov, V. N.; Keith, T.; Kobayashi, R.; Normand, J.; Raghavachari, K.; Rendell, A.; Burant, J. C.; Iyengar, S. S.; Tomasi, J.; Cossi, M.; Rega, N.; Millam, J. M.; Klene, M.; Knox, J. E.; Cross, J. B.; Bakken, V.; Adamo, C.; Jaramillo, J.; Gomperts, R.; Stratmann, R. E.; Yazyev, O.; Austin, A. J.; Cammi, R.; Pomelli, C.; Ochterski, J. W.; Martin, R. L.; Morokuma, K.; Zakrzewski, V. G.; Voth, G. A.; Salvador, P.; Dannenberg, J. J.; Dapprich, S.; Daniels, A. D.; Farkas, O.; Foresman, J. B.; Ortiz, J. V.; Cioslowski, J. and Fox, D. J. Gaussian, Inc., Wallingford CT, **2013**.

[S3] Schleyer, P. v. R.; Maerker, C.; Dransfeld, A.; Jiao, H.; Hommes, N. J. R. v. E. *J. Am. Chem. Soc.* **1996**, *118*, 6317.

[S4] Geuenich, D.; Hess, K.; Köhler, F.; Herges, R. *Chem. Rev.* **2005**, *105*, 3758.
